# Geochemical characteristics of strontium isotopes in a coastal watershed: implications for anthropogenic influenced chemical weathering and export flux

**DOI:** 10.7717/peerj.13223

**Published:** 2022-04-05

**Authors:** Shitong Zhang, Guilin Han, Jie Zeng

**Affiliations:** Institute of Earth Sciences, China University of Geosciences (Beijing), Beijing, China

**Keywords:** Sr isotopes, Silicate weathering, Coastal river, Human activities, SE China

## Abstract

Coastal watershed are essential in transporting dissolved loads from terrestrial biogeochemical process of surface environment to the adjacent oceans. The solute chemistry of coastal river water contains significant information about environmental processes under the impact of both natural lithology and anthropogenic pressure. In this study, strontium (Sr) isotopes and water chemistry data of the Jiulongjiang (JLJ) river water were analyzed in detail to trace the contribution of bedrock weathering, and quantify Sr flux to the East China Sea (ECS). The dissolved Sr contents ranged 0.07–0.90 μmol L^−1^ and greatly fluctuated where tributaries encountered, and ^87^Sr/^86^Sr values relatively fluctuated between 0.7140 and 0.7514. Silicate weathering was identified to be the predominant contribution of riverine dissolved loads. Strontium flux to the ocean in dry season was estimated to be 689.2 tons per year, implying an essential influence on oceanic strontium evolution. In accordance with forward model, the silicate weathering rate and CO_2_ consumption rate were 55.7 tons km^−2^ per year and 16.9 × 10^5^ mol km^−2^ per year, respectively, slightly higher than world average. Considering anthropogenic impacts alongside the river, the integrated effect of lower runoff and longer retention time of river water in dry season may aggravate weathering processes. Although CO_2_ sink by silicate weathering in JLJ seems less than the sink in world’s central reservoirs, it should still be taken into consideration for coastal carbon budget. These findings highlight the use of geochemical characteristics of strontium and its isotopes in identifying weathering process and output flux to the ocean, which provides basic data for sustainable coastal water resource management.

## Introduction

As a major input source to the adjacent ocean, coastal watershed is commonly considered as a significant place to investigate environmental biogeochemical processes through river-ocean transportation (Gaillardet et al., 1999). From the view of two typical categories in terrestrial chemical weathering (carbonate/silicate weathering), CO_2_ consumption *via* carbonate weathering is generally regarded as a temporary sink, since the CO_2_ flux consumed by carbonate dissolution could be balanced *via* carbonate precipitation from ocean during geological timescale (Cao et al., 2020; Stevenson et al., 2018). However, continental weathering of silicate rocks is recognized as CO_2_ net sink in the carbon cycle over 1 million years, since it separates and releases net carbon from the atmosphere to coastal rivers which finally to the adjacent sea (Li et al., 2021; Moon et al., 2007). Therefore, the typical costal watershed contained mixing carbonate-silicate lithology is significant to distinguish these two weathering proportions, and also essential in assessing the net CO_2_ sink *via* silicate weathering (Mortatti & Probst, 2003). Numerous research interests on riverine geochemistry have been largely attracted to weathering processes and their geological implications (Gaillardet et al., 1999; Moon et al., 2007; Roy, Gaillardet & Allegre, 1999).

The variation of isotope abundances in Earth surface provides a tracing method for chemical processes which have been generated through geologic time (Han et al., 2020). Isotope tracing offers an extra insight on tracking specific elements, thus it is an increasingly important technique in environmental studies (Capo, Stewart & Chadwick, 1998). Considering the analytical advances of non-traditional stable isotope systems in two decades, strontium isotopes (^87^Sr/^86^Sr) have been widely applied as a powerful tool in understanding basic earth processes and evolution (Négrel & Deschamps, 1996; Palmer & Edmond, 1989), since natural biogeochemical processes do not generate obvious Sr isotopic fractionation (Krishnaswami et al., 1992; Singh, Kumar & France-Lanord, 2006). Strontium is primarily released by rock weathering and cycled *via* plants and animals, which finally enters the ocean reservoir mainly through coastal rivers (Capo, Stewart & Chadwick, 1998). Furthermore, climate change and tectonic activities could alter terrestrial weathering intensity and the dominant rock types of weathering to a certain degree (Boral et al., 2021; Liu et al., 2019b), which indirectly produces indispensable effect on the amount of Sr and its isotopes entering the oceans through coastal rivers. Therefore, compared to traditional riverine hydro-geochemistry and other metal isotopes, Sr isotopes are far more sensitive in weathering source identification and proven as an essential tracer for continental process coastal rivers around the world (Gaillardet et al., 1999; Millot et al., 2002; Xu et al., 2011).

From another perspective, given that marine ^87^Sr/^86^Sr record is one of the best reconstructed isotope records in seawater (Boral et al., 2021), the strontium isotopic composition of coastal river provides significant insight on elemental fluxes to the ocean, which is essential for illustrating marine isotope records (Stevenson et al., 2018). Previous researches have demonstrated that the quick seawater ^87^Sr/^86^Sr ratio rise during the last 2 Ma is primarily attributed to the alteration of strontium entering the sea from coastal watershed (Capo, Stewart & Chadwick, 1998; Peucker-Ehrenbrink & Fiske, 2019). Therefore, dissolved Sr flux in a coastal watershed could reveal the potential Sr isotope evolution in the ocean (Zieliński et al., 2018). Although previous studies have already explored numerous hydro-geochemical and isotopic characteristics of the world largest rivers (Gaillardet et al., 1999; Mortatti & Probst, 2003; Singh, Kumar & France-Lanord, 2006; Yang et al., 2021; Zeng & Han, 2020; Zeng, Han & Yang, 2020), further systematic investigations of Sr isotopic geochemical characteristics in typical coastal watershed are still in urgent need to be accurately constrained for better understanding of marine records.

The Jiulongjiang (JLJ) River is a typical large coastal river which flows from southeast (SE) China and finally transports terrestrial materials to the South China Sea (Liu & Han, 2020). Due to the mixing lithology of both silicate and carbonate bedrocks, the JLJ River basin is considered as an appropriate place to investigate the contribution and implication of the two weathering process. In addition, the rapid development of industry and agriculture within the river basin has made huge impact on regional environment. Superimposed on natural processes, the anthropogenic inputs such as nitrogen fertilizers, acid mine wastewaters, industrial effluents and urban sewage give rise to the variation of chemical compositions in the river water (Li et al., 2019; Zeng et al., 2022). Besides the major weathering agent of carbonic acid, nitric acid and sulfuric acid produced by human inputs may also help accelerate chemical weathering by the additional release of the proton to river water (Li et al., 2008; Xu et al., 2021). Therefore, the anthropogenic pressure on the typical coastal watershed should also be considered when assessing the contribution of chemical weathering on dissolved ions.

Here, Sr and its isotope geochemistry of the JLJ river water is systematically investigated along with previous water chemistry data to: (a) clarify the spatial distribution of dissolved strontium concentration and its isotopic ratios; (b) discriminate the constraints and contributions of weathering process and estimate Sr output flux; (c) estimate silicate weathering rate and CO_2_ uptake within the coastal watershed. This study is essential to shape a geochemical view of coastal riverine dissolved loads and beneficial for the local water resource management.

## Materials and Methods

### Study area and geological setting

**Figure 1:**
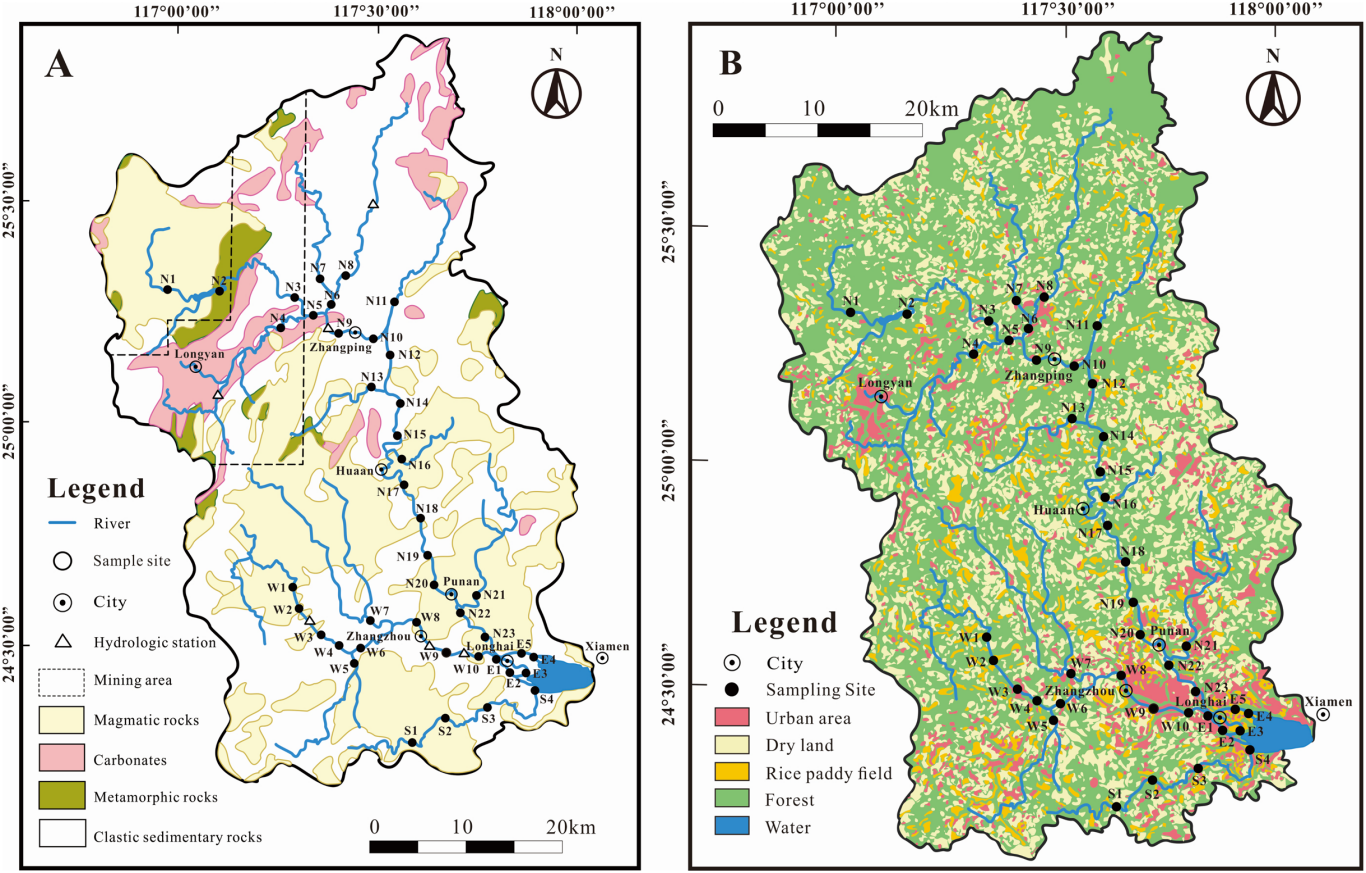
Sketch map in the JLJ River basin with sampling sites. (A) Bedrock lithology; (B) land-use types.

The Jiulongjiang (JLJ) river originates from the southeast edge of the Eurasia Plate in the Fujian Province, SE China (24°18′–25°88′N, 116°78′–118°03′E) and flows all the way from the NW-SE trending of 260 km through the Xiamen Bay to the South China Sea (Fig. 1) (Li et al., 2019; Liang et al., 2018). The drainage area of the JLJ river is 14,087 km^2^ with a mean water discharge of 14 km^3^ per year. North river (NR, mainstream), West river (WR, main tributary) and South river (SR, main tributary) are three main channels in the JLJ river, which predominantly flow over cretaceous A and I-type granites (98–119 Ma) with a relatively small amount of Jurassic clastic rocks and carbonates (Liu & Han, 2020). A bit of sandstone is also distributed along the estuary with no obvious existence of evaporites.

The JLJ River basin is attributed to subtropical oceanic monsoon climate together with high seasonal fluctuations of regional temperature, precipitation and runoff. Average temperature over the JLJ River basin ranges between 19.9 °C in winter and 21.1 °C in summer (Liu & Han, 2021a). The majority of rainfall (more than 80%) is concentrated from April to September (wet season), which exhibits the annual rainfall of 1,400–1,800 mm per year. The detailed description of terrain and hydrology can be found through our previous work (Li et al., 2019; Liu & Han, 2021a; Yang et al., 2018).

The upper part of the JLJ are situated in mountainous areas which has high vegetation coverage and less anthropogenic impact, while the middle-lower part are adjacent to rural and urban areas which are highly influenced by anthropogenic activities (Fig. 1) like industrial facilities, substantial deposits/mining, and urbanization (Li et al., 2019). Due to extensive agricultural and industrial development for >40 million residents, the hydrochemistry have been impacted by a great amount of anthropogenic perturbations, including the construction of many cascade reservoirs on lower reaches and coal mining activities in the upper basin (Liu & Han, 2021a).

### Sample collection

A total of 42 water samples were collected from downstream to upstream in January 2018 (dry season) along the JLJ River basin. The acid-washed high density polyethylene (HDPE) bottle was used to load samples from different lithology, land use and hydrological conditions. Water samples were labelled with N1–N23 for 23 samples from North River (NR), W1–W10 for 10 samples from West River (WR), S1–S4 for four samples from South River (SR), and E1–E5 for five samples from the estuary (E), respectively. All samples were filtered through a 0.22-μm membrane (Millipore). For anion measurement, the samples were kept in HDPE bottle directly, whereas for cations, samples were acidified by HCl in high purity until pH <2. Containers were then sealed and kept at 4 °C for further analysis. The detailed information of sampling procedures has been documented through our previous works (Liu & Han, 2021b; Zeng, Han & Zhu, 2019).

Following the pretreating procedure for Sr isotope analysis (Li & Han, 2021), after the concentration through evaporation, the samples for isotope measurement were separated from other ions through a standard ion-exchange technique. Sr isotope were analyzed by a Nu Plasma 3 multi-collector mass spectrometer (MC-ICP-MS; Nu Instruments, Wrexham, UK) at the Surficial Environment and Hydrological Geochemistry Laboratory in the China University of Geosciences, Beijing. In order to correct mass bias, the ^87^Sr/^86^Sr ratios were normalized by a constant ^86^Sr/^88^Sr value of 0.1194 internally with the exponential law. The evaluation of accuracy in our calculation were monitored periodically *via* the standard solution NIST SRM 987 with a mean ^87^Sr/^86^Sr ratio of 0.710276 ± 33 (2SD, *N* = 46).

## Methodology

The source apportionment analysis of Sr isotope is based on the mass conservation equations as follows:

(1) $${\left( {\displaystyle{{{}_{}^{{\rm 87}}{\rm Sr}} \over {{}_{}^{{\rm 86}}{\rm Sr}}}} \right)_{{\rm riv}}} = {\left( {\displaystyle{{{}_{}^{{\rm 87}}{\rm Sr}} \over {{}_{}^{{\rm 86}}{\rm Sr}}}} \right)_{{\rm rain}}}{\rm \times \; S}{{\rm r}_{{\rm rain}}} + {\left( {\displaystyle{{{}_{}^{{\rm 87}}{\rm Sr}} \over {{}_{}^{{\rm 86}}{\rm Sr}}}} \right)_{{\rm carb}}}{\rm \times \; S}{{\rm r}_{{\rm carb}}} + {\left( {\displaystyle{{{}_{}^{{\rm 87}}{\rm Sr}} \over {{}_{}^{{\rm 86}}{\rm Sr}}}} \right)_{{\rm evap}}}{\rm \times \; S}{{\rm r}_{{\rm evap}}} + {\left( {\displaystyle{{{}_{}^{{\rm 87}}{\rm Sr}} \over {{}_{}^{{\rm 86}}{\rm Sr}}}} \right)_{{\rm sili}}}{\rm \times \; S}{{\rm r}_{{\rm sili}}}\; \; \;$$

Here, the (^87^Sr/^86^Sr)_riv_, (^87^Sr/^86^Sr)_rain_, (^87^Sr/^86^Sr)_carb_, (^87^Sr/^86^Sr)_evap_ and (^87^Sr/^86^Sr)_sili_ means the ^87^Sr/^86^Sr values of the JLJ river water, rainfall, carbonate, evaporite and silicate bedrocks, respectively. The proportions of relative contribution in the end-members above are represented by Sr_rain_, Sr_carb_, Sr_evap_ and Sr_sili_. Furthermore, the silicates weathering rates (SWR) can be calculated by cations from silicate weathering (either by H_2_CO_3_ or H_2_SO_4_), and the silicate weathering derived-CO_2_ consumption rate (ФCO_2_-Sili) can be estimated through charge balance (the unit of cation is mol L^−1^) from the following equations (Moon et al., 2007):

(2) $$\rm SWR = {(K_{sili} + Na_{sili} + Ca_{sili} + Mg_{sili} + SiO_{2}) \times Discharge/Area}$$

(3) $${\rm{\Phi C}}{{\rm{O}}_2} - {\rm{Sili}} = {\rm{\Phi T}}{{\rm{Z}}^ + } - {\rm{Sili}} = ({{\rm{K}}_{{\rm{sili}}}} + {\rm{N}}{{\rm{a}}_{{\rm{sili}}}} + 2{\rm{C}}{{\rm{a}}_{{\rm{sili}}}} + 2{\rm{M}}{{\rm{g}}_{{\rm{sili}}}}) \times {\rm{Discharge}}/{\rm{Area}}$$

In addition, the strontium flux of the JLJ river is estimated *via* the equation as follows (Wang, Zhang & Liu, 2007):

(4) $$\rm F_{Sr} = Q_{JLJ} \times C_{Sr}$$

Here, F_Sr_ represents the Sr flux in the JLJ, Q_JLJ_ represents the average river runoff (in m^3^ s^−1^), and C_Sr_ represents the mean content of dissolved Sr (in mg L^−1^). All analysis were processed by Microsoft Office 2013 and SPSS 26.0 (IBM Corporation, Armonk, NY, USA). The Origin 2017 and CorelDraw 2018 software were used to edit graphs.

## Results

### Water physicochemical parameters

**Figure 2:**
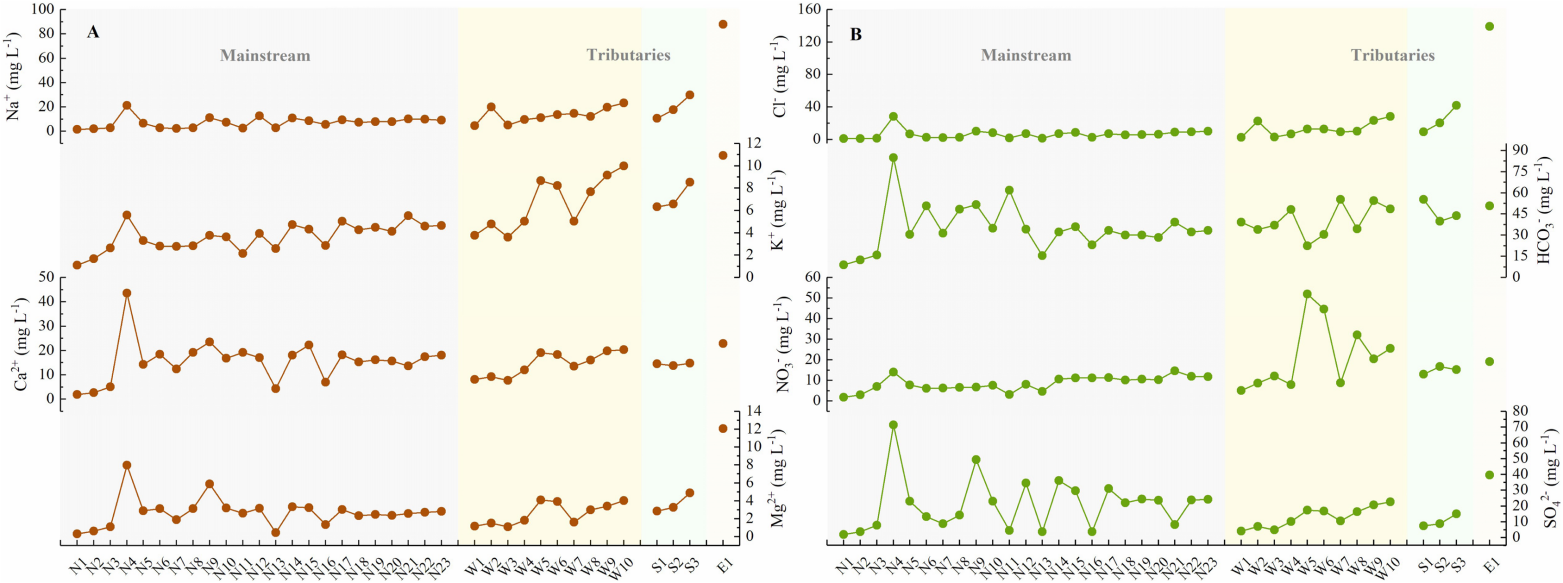
The concentrations of physicochemical parameters in the JLJ River water and their variations from the upper to lower reaches. (A) Na^+^, K^+^, Ca^2+^, and Mg^2+^; (B) Cl^−^, HCO_3_^−^, NO_3_^−^, and SO_4_^2−^.

The general physicochemical parameters in the JLJ river water are shown in Table S1 and Fig. 2. The water temperature appeared to be in the range of 11.0 and 22.7 °C (average 16.4 °C). As an indicator of comprehensive water quality, river pH plays a significant role in altering the occurrence and transformation of biological elements. The water was mildly acidic to alkaline ranged from 6.3 to 8.8 (average 7.2), evenly distributed across the whole basin. As for the spatial variation, pH in both tributaries (WR and SR) displayed an increasing trend along the river flow, while the mainstream (NR) showed fluctuated variation. It is noteworthy that the site near N15 and N21 shows relatively high pH, indicating the impact from the adjacent urban areas (Huaan and Punan).

Pearson correlation matrix was applied to investigate the relationships of hydro-chemistry ionic species (which belongs to continuous variable) in the JLJ watershed. Bilateral test was performed to analyze the significance of 36 sample sizes. The corresponding test statistics and the exact *p*-value were exhibited in Tables S2 and S3. As shown in Table 1, EC and the total dissolved solids (TDS) strongly correlated with each other (R = 0.99). The average values of total dissolved solids (TDS) and EC contents were 119.5 mg L^−1^ and 227.3 μS cm^−1^, respectively, and the average value of TDS in NR (94.9 mg L^−1^) is much lower than that in WR (124.4 mg L^−1^) and SR (142.6 mg L^−1^). Some samples near the estuary displayed unusual values of TDS (≥5,000 mg L^−1^) due to the mixing of seawater, thus was excluded from our studies. Compared with other rivers, the average TDS in JLJ River water is much lower than that in the Yangtze River (557 mg L^−1^) (Chetelat et al., 2008), but is similar to that in rivers draining through silicate areas, such as Parana (86 mg L^−1^) and Uruguay (60 mg L^−1^) (Gaillardet et al., 1999).

**Table 1:** Correlation coefficient (R) matrix between hydro-chemistry ionic species (in mg L^−1^) in JLJ river water.

	pH	EC	Na^+^	K^+^	Ca^2+^	Mg^2+^	Cl^−^	NO_3_^−^	SO_4_^2−^	HCO_3_^−^	SiO_2_	TDS	Sr
pH	1												
EC	0.173	1											
Na^+^	0.057	0.836**	1										
K^+^	0.082	0.740**	0.808**	1									
Ca^2+^	0.333*	0.841**	0.454**	0.396*	1								
Mg^2+^	0.283	0.912**	0.608**	0.517**	0.911**	1							
Cl^−^	0.099	0.821**	0.963**	0.762**	0.460**	0.636**	1						
NO_3_^−^	0.038	0.477**	0.424*	0.789**	0.281	0.382*	0.389*	1					
SO_4_^2−^	0.108	0.731**	0.377*	0.208	0.848**	0.814**	0.348*	0.101	1				
HCO_3_^−^	0.247	0.673**	0.453**	0.316	0.742**	0.658**	0.447**	−0.002	0.451**	1			
SiO_2_	−0.073	0.244	0.517**	0.609**	−0.149	0.039	0.456**	0.465**	-0.362*	0.185	1		
TDS	0.174	0.990**	0.841**	0.787**	0.824**	0.899**	0.817**	0.565**	0.685**	0.653**	0.318	1	
Sr	0.053	−0.21	−0.174	0.005	−0.227	−0.138	−0.147	0.207	−0.257	−0.336*	0.071	−0.179	1

**Notes:**

* Correlation is significant at *p* < 0.05 level.

** Correlation is significant at *p* < 0.01 level.

The total cationic charges (TZ^+^) and anionic charges (TZ^−^) commonly remain balanced in natural unpolluted water. TZ^+^ varied from 0.2 to 6.2 meq L^−1^ in JLJ river water (average 1.7 meq L^−1^) during sampling period, slightly higher than world average (1.25 meq L^−1^) (Martin & Meybeck, 1979), indicating the influence from the geological bedrock types and distributions. The normalized inorganic charge balance (NICB = (TZ^+^ − TZ^−^)/TZ^+^) of most samples were <10%, whereas site N15 and W3 displayed abnormal NICB values (+12.0% and −22.9%), which may be attributed to the ion release from the discharge of urban sewage and wastewater within the watershed.

Our previous work have demonstrated the dominant species of Ca^2+^ and HCO_3_^−^ followed by Na^+^ and SO_4_^2−^ in JLJ River waters (Li et al., 2019). HCO_3_^−^ greatly fluctuated from 8.8 to 84.9 mg L^−1^ along the flow, and the NR draining carbonate areas (near site N4) displayed extremely high HCO_3_^−^ values. SO_4_^2−^ ranged from 2.0 to 71.5 mg L^−1^ (average 18.6 mg L^−1^) with generally higher values in NR than that in WR and SR, which may ascribe to the coal-bearing stratum in the upper reach of NR due to no appearance of evaporites. Besides, it is worth noting that the sites near N9 had much higher SO_4_^2−^ concentration, which may result from the anthropogenic impacts from the adjacent urban area (Zhangping City) according to the geological map (Fig. 1).

Major dissolved riverine ions mainly originate from atmospheric deposition, anthropogenic input and bedrock weathering (Wang, Zhang & Liu, 2007). The influence of rainfall during our sampling period is limited since the JLJ River was still in dry season. Thus, silicate weathering is suggested to dominate the dissolved loads in the JLJ River water based on regional lithology (Fig. 1), although the predominant cation seemed to be Ca^2+^ rather than Na^+^ and K^+^. This phenomenon may be ascribed to the lower river-flow rate in dry season (winter) which could benefit water-rock interactions and thus enhance the weathering of limited carbonates in the upper reach of NR. In addition, previous study has also indicated that carbonates could weather 10 to 20 times faster than silicate rocks based on its sensitivity to climate changes and human perturbations (Gaillardet et al., 2018). In the next session, the application of Sr isotopes could help constrain the contributions from the process of chemical weathering.

### Spatial variation of Sr and its isotopes

It is of primary interest to determine the variation and its implication of dissolved Sr within the watershed, which requires an investigation in the geochemical characteristics of Sr together with the relationship of water chemistry. As exhibited in Table 2, the dissolved Sr concentration in the JLJ River widely fluctuated from 0.07 to 0.90 μmol L^−1^ (average 0.33 μmol L^−1^), lower than the global average of 0.89 μmol L^−1^ (Palmer & Edmond, 1992). The value of Sr concentration in JLJ River water showed no significant trend along the river flow, and the average Sr contents in WR (0.28 μmol L^−1^) is mildly lower than that in NR (0.32 μmol L^−1^) and SR (0.39 μmol L^−1^). It is worth noting that the measured dissolved Sr contents greatly fluctuated where tributaries encountered (site N4–N5, N11–N12, N21–N22, W5–W8) within the watershed. Moreover, site N4 draining through carbonate areas exhibited much lower Sr concentration values (0.007 mg L^−1^) than we expected, which probably be ascribed to the disruption by water mixing process. According to regional lithology (Fig. 1), site N2 draining through metamorphic rocks exhibited much higher Sr values than the adjacent sampling sites. Besides, the Sr contents of site N12, N22 and W9–W10 may get a huge impact from their neighboring cities, and the adjacent seawater mixing process may also play a potential role in water samples from the estuary (E). Compared with the previously reported Sr data of 1.38 μmol L^−1^ in average in the dry season of JLJ River 10 years ago (year 2008) (Zhou et al., 2012), the dissolved Sr concentration of this study sampled in 2018 (average 0.33 μmol L^−1^) is much lower. Considering that the sampling sites of previous study were adjacent to the river estuary, and that the Sr composition in the estuary (E1–E5) of this study merely varied between 0.39 μmol L^−1^ and 0.61 μmol L^−1^, this concentration divergence may be attributed to the variable impact from seawater intrusion, since the estuary is greatly affected *via* seawater intrusion and mixing process, especially in dry season (Zhou et al., 2012).

**Table 2:** Statistical data of strontium concentrations (in μmol L^−1^) and ^87^Sr/^86^Sr ratios of the river water.

Parameters	Min	Max	Mean	Median	SD	CV
Sr-Mainstream NR (μmol L^−1^)	0.08	0.9	0.32	0.30	0.184	0.58
Sr-Tributary WR (μmol L^−1^)	0.07	0.54	0.28	0.26	0.162	0.60
Sr-Tributary SR (μmol L^−1^)	0.34	0.45	0.39	0.38	0.052	0.13
Sr-Estuary (μmol L^−1^)	0.39	0.61	0.47	0.44	0.088	0.19
Sr-JLJ whole basin (μmol L^−1^)	0.07	0.90	0.33	0.32	0.168	0.50
Sr-Global average (μmol L^−1^) (Palmer & Edmond, 1992)			0.89			
^87^Sr/^86^Sr-JLJ whole basin	0.7140	0.7514	0.7357	0.7357	0.010	0.01
^87^Sr/^86^Sr-Global average (Palmer & Edmond, 1992)			0.7119			
^87^Sr/^86^Sr-Seine River (Roy, Gaillardet & Allegre, 1999)	0.7077	0.7168				
^87^Sr/^86^Sr-Indus River (Karim & Veizer, 2000)	0.7098	0.7120				
^87^Sr/^86^Sr-Himalayan (Oliver et al., 2003)	0.7115	0.9646				
^87^Sr/^86^Sr-W. Greenland (Goldstein & Jacobsen, 1987)	0.826	0.943				

**Figure 3:**
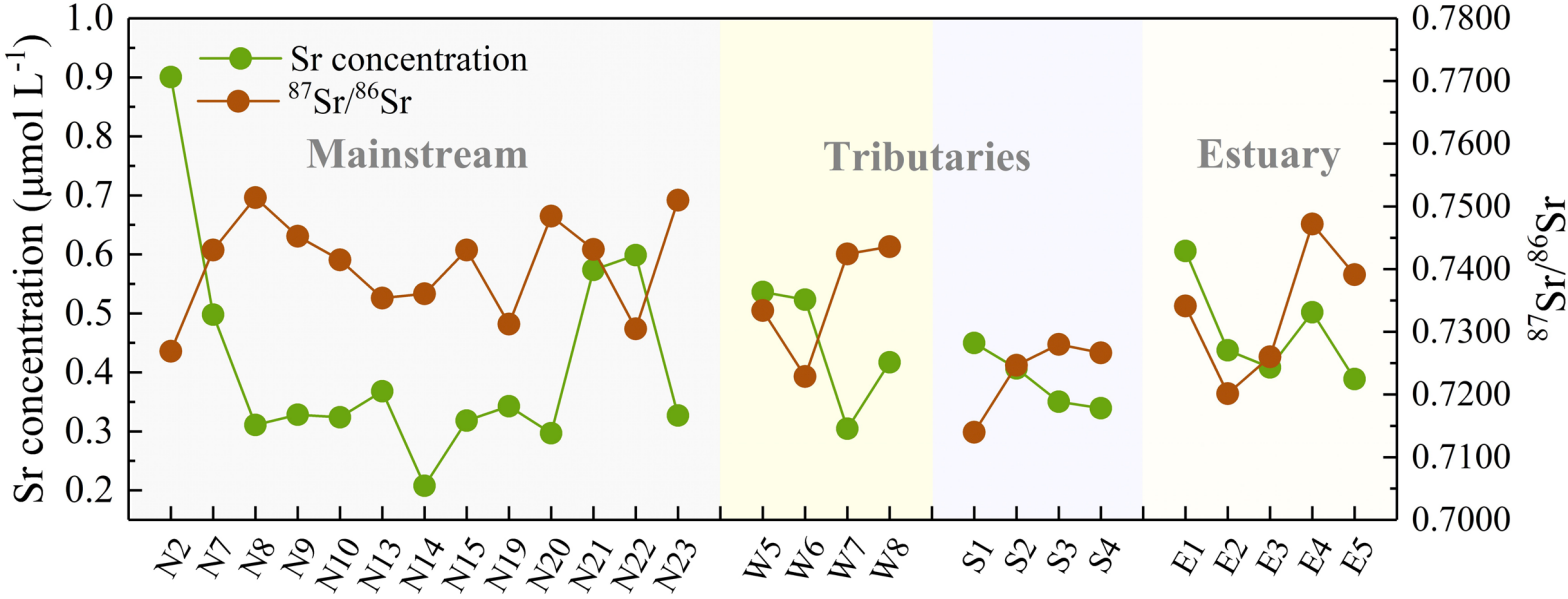
Downstream variations in Sr concentration and its isotopic composition of the JLJ River.

The JLJ River water was characterized by relatively fluctuated ^87^Sr/^86^Sr ratios between 0.7140 and 0.7514 with an average of 0.7357 (Table S1 and Fig. 3), much higher than that in Seine River of France (0.7077–0.7168) (Roy, Gaillardet & Allegre, 1999) and Indus River (0.7098–0.7120) (Karim & Veizer, 2000), but lower than that in rivers draining though Himalayan (0.7115–0.9646) (Oliver et al., 2003) and the 3.8-Ga-old Isua region of W. Greenland (0.826–0.943) (Goldstein & Jacobsen, 1987). The average value of Sr isotopic ratios is higher than world average of 0.7119 (Palmer & Edmond, 1992). Moreover, Sr isotopic compositions in the SR was relatively lower than that in NR and WR, as shown in Fig. 3.

## Discussion

### Geochemical implications of dissolved ^87^Sr/^86^Sr

**Figure 4:**
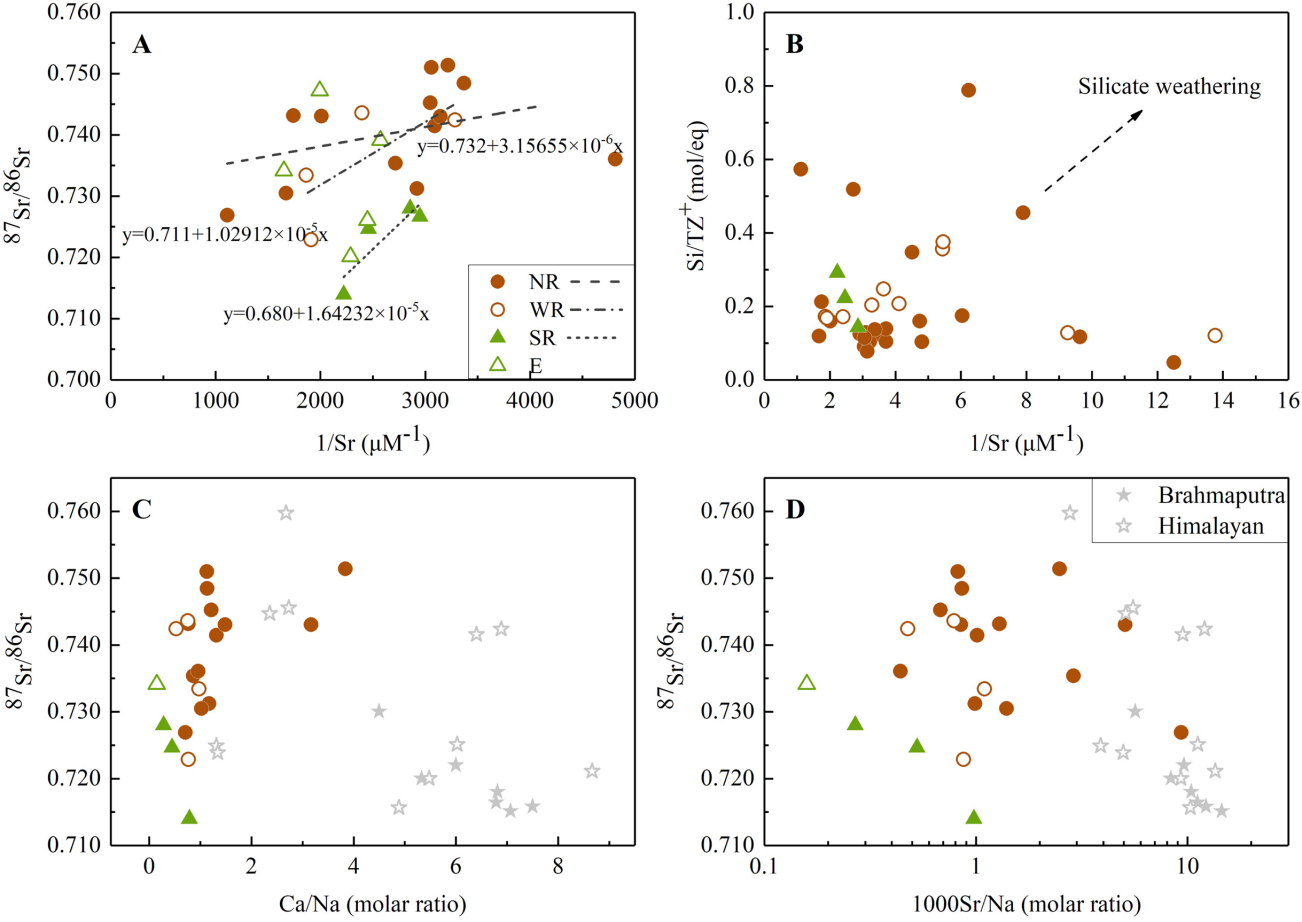
The relationships between Sr isotope and elemental ratios. (A) Relationship between 1/Sr and ^87^Sr/^86^Sr ratios in the JLJ River basin; (B) 1/Sr *vs* Si/TZ^+^ ratios in the JLJ watershed; (C) Mixing diagrams using Ca/Na molar ratios and Sr isotopic ratios in the JLJ River waters; (D) ^87^Sr/^86^Sr *vs* 1000Sr/Na mixing diagrams within the JLJ River basin. The composition data of Brahmaputra and Himalayan river water were derived from the literature (Singh, Kumar & France-Lanord, 2006).

Normally, variations of riverine Sr concentrations and ^87^Sr/^86^Sr ratios are largely discriminated by two distinct geochemical pathways: one component having higher strontium content with lower ^87^Sr/^86^Sr ratios (more typical of carbonate weathering), and vice versa for river draining silicate bedrocks (Krishnaswami et al., 1992; Palmer & Edmond, 1992). Fig. 4A exhibited ^87^Sr/^86^Sr and 1/Sr plot in the dissolved loads of the JLJ mainstream (NR), two tributaries (WR and SR) and the estuary (E). The positive relationships existed between 1/Sr and ^87^Sr/^86^Sr ratios, and this linear trend displayed in both main channel and two tributaries, suggesting an explanation in terms of mixing process similar as narrated above. The ^87^Sr/^86^Sr ratios in most samples (Table S1 and Fig. 4) were higher than those in carbonates (0.706–0.709) (Krishnaswami et al., 1992), implying potential impact of Sr isotopic input by other weathering materials. According to regional lithology (Fig. 1), silicate weathering is suggested to dominate the solutes in the JLJ River, although the predominant cation seems to be Ca^2+^ rather than Na^+^ and K^+^. This phenomenon may be ascribed to the lower river-flow rate in dry season (winter) which could benefit water-rock interactions and thus enhance the weathering of limited carbonates in the upper reach of NR. In addition, previous study has also indicated that carbonates could weather 10 to 20 times faster than silicate rocks based on its sensitivity to the climate changes and human perturbations. However, the weathering of silicate bedrock may also exert essential constraints on radiogenic Sr isotope ratios, since a systematic raise of dissolved Si contents were observed from the upper reach of NR to the two main tributaries (WR and SR). This suggested the increasing contribution of silicate additions along the river flow, especially in WR and SR where only limited carbonates existed, which is also consistent with the plotting of 1/Sr and Si/TZ^+^ ratios in the JLJ watershed (Fig. 4B).

On the plot of Fig. 4A, sample values dispersed to higher Sr isotopic ratio rather than following a single mixing trend, suggesting a mixing of multi isotopic end-members along the JLJ stretch. According to previous study (Roy, Gaillardet & Allegre, 1999), water draining carbonates could be discriminated by higher molar ratios of Sr/Na and Ca/Na (even greater than 50.10^−3^ and 50), while those draining through silicate bedrocks could be identified by much lower ratios (0.5^−1^ for Ca/Na and 3.10^−3^ for Sr/Na). As indicated in Figs. 4C and 4D, most water samples in JLJ River had much lower elemental molar ratios (0–2 for Ca/Na and near 1 for 1000Sr/Na) approaching to Brahmaputra and Himalayan rivers which derived their radiogenic Sr mostly from silicates (Singh, Kumar & France-Lanord, 2006), but appeared away from the value of carbonates mentioned above. Therefore, the impact of silicate weathering in the JLJ watershed is proved and would be further quantified based on the mass-balance model.

### Sources and weathering contributions of dissolved Sr

Riverine strontium isotopes do not fractionate during weathering and other biogeochemical processes, but exhibit identical isotopic signatures when draining various types of bedrock lithology, thus have been widely applied to hydro-chemical researches for identifying weathering provenances within a drainage (Han, Tang & Xu, 2010; Roy, Gaillardet & Allegre, 1999). Quantifying and discriminating the amount of Sr originated from silicates and carbonates is essential to estimate the abundance of silicate-derived Sr flux which could correspondingly alter regional climate through sufficient soild-Earth CO_2_ degassing (Wang, Zhang & Liu, 2007). The relative contributions of carbonate and silicate weathering on riverine strontium isotopic ratios can be determined as Eq. (1).

The average value of ^87^Sr/^86^Sr in carbonate river water and rainwater were close to 0.709 based on previous research (Han & Liu, 2004). From a more accurate perspective, the degree of water-rock interaction within the basin, especially the alteration of relative mineral weathering rate in multi-mineral rock system, could affect the Sr isotopic composition of river water to some extent (Rao et al., 2020). Therefore, the strontium isotope ratios in river water should originate from the isotopic values of Sr-bearing minerals in the basin rather than the whole rock types. However, due to lack of isotopic data for regional carbonates and sample sites near carbonate outcrop, the Sr-bearing minerals of regional carbonates was not clear. Therefore, the commonly referenced value of 0.709 in carbonate rivers (Han & Liu, 2004) was selected to represent the end-member value from carbonates. The segment of (^87^Sr/^86^Sr)_evap_ was neglected because of no evaporate distribution in the JLJ basin (Fig. 1). For silicate segment, research have demonstrated that the Sr geochemical behavior in granitic (silicate) systems shows a coherence with Ca (Chen et al., 2020), and Sr prefers to concentrate in late crystallization phases. A previous work on Sr isotopes of pegmatite near our study area in Fujian Province, southeast (SE) China indicated that the “primary Sr minerals” of K-feldspar and apatite (Rao et al., 2020). Therefore, the ^87^Sr/^86^Sr ratio of feldspar (average 0.7341) and apatite (average 0.7300) in silicate rocks of this region (Rao et al., 2020) should be representative for analysis. However, the relative weight of these two representative minerals was not clear. From another perspective, according to the recent study of riverine sediments (Yu et al., 2019), ^87^Sr/^86^Sr values of surface sediments in the JLJ River (0.7141–0.7335) were similar to that of Fujian granite (0.7094–0.7520) and Western Fujian magmatic rock (0.7133–0.7412) (Huang et al., 1986; Lin, Shen & Huang, 1999), indicating the major sediment provenance of local natural (especially upstream) granite and magmatic rocks. Considering that the Sr isotopic ratios of measured water samples were relatively high, the maximum ^87^Sr/^86^Sr value of 0.7334 in the regional surface sediments in previous riverine sediment research (Yu et al., 2019) was thought to be the most representative for the segment of (^87^Sr/^86^Sr)_sili_. This value was also in accordance with the range of Sr-bearing minerals in regional silicate rock (Rao et al., 2020). Even so, some measured values still exceeded this figure (0.7334), which may be attributed to uncertain inputs from anthropogenic activities. Therefore, the samples exhibiting higher values were not included in our calculation.

Together with the mixing equation (Sr_rain_ + Sr_carb_ + Sr_sili_ = 1), the conclusion can be drawn that the proportion of silicate weathering explained more than half of total dissolved Sr in the JLJ River basin (average 66%, ranged 20.4% to 91.1%), while the segment of rainwater and carbonate weathering occupied the other 34% (ranged 8.9% to 79.6%). For the JLJ mainstream (NR), generally, silicate weathering could interpret 73.3–91.1% of dissolved Sr in the river, while carbonate and rainwater accounted for the other 8.9–26.7%. Due to lack of measured strontium isotopic data near sampling site N4 where the only carbonates outcropped within the scope of study area (Fig. 1), our calculation may leave open the possibility of deviation to some extent. For the two main tributaries (WR and SR), silicate weathering contributed 56.8% of dissolved Sr in WR water samples, and 20.4–77.9% in SR. The silicate weathering contribution proportions of dissolved Sr in the estuary exhibited 45.7–69.8%, and 30.2–54.3% by carbonates and rainwater, which may also get an impact from the adjacent seawater mixing process. In general, the results significantly indicated that the JLJ River basin is predominated by silicates from magmatic rocks and local granite *via* natural geological processes.

### Silicate weathering and CO_2_ consumption under human impact

Rock weathering consumes atmospheric CO_2_ and makes great contribution to the long-term CO_2_ evolution, thus further alters global temperature of the surficial environment (Roy, Gaillardet & Allegre, 1999). Therefore, the analysis of chemical weathering rates and CO_2_ uptake help reveal the weathering process within the coastal river and the impact on its adjacent ocean. Dissolved phases in the watershed mainly reflect sources from bedrock lithology (silicate and carbonate weathering), atmosphere deposition, and anthropogenic activities (mostly urban sewage and agricultural impact). On the contrary to natural rivers like Amazon and Congo, the JLJ is apparently impacted by anthropogenic activities. In order to reduce the sensitivity of the hydro-chemistry on thin surficial deposits, we applied the forward model to investigate major ions and isotopes to the JLJ River from their potential sources during dry season (Gaillardet et al., 1999; Moon et al., 2007), that is, assuming a mixing model between proportions of water mass and pre-assigned chemical parameter signatures. The accuracy of this model mostly depends on the pertinent choice of end-member constraints (Negrel et al., 1993).

The sampling sites of N23, W10 and S3 were selected to represent the ion derived from NR, WR and SR, as they are located at the most downstream channels without tidal influence. Considering that no Cl is obtained from evaporites, the Cl content of water samples and normalized ratios of rainwater were applied to make corrections for atmospheric inputs based on following equation (Moon et al., 2007):

(5) $$\rm X_{rain} = (X/Cl)_{rain} \times Cl_{min}$$

Here, X denotes the major riverine cations (K, Na, Ca, Mg); (X/Cl)_rain_ stands for the element ratios of local rainwater; and Cl_min_ represents the lowest Cl concentration of the JLJ River waters with the assumption that Cl_min_ is originated totally from rainwater. Rainwater Cl-normalized ratios in the JLJ River basin (average K/Cl = 0.2, Na/Cl = 0.9, Ca/Cl = 1.1, Mg/Cl = 0.2) were referenced from previous work (Li et al., 2019). Besides, the Cl concentration of site N1 exhibited the lowest value (1.1 mg L^−1^), thus was regarded as Cl_min_ in the calculation.

Human inputs could alter natural weathering process through the addition of sulfuric acid mainly from mining activities and nitric acid from agriculture. These extra protons together with carbonic acid accelerate chemical weathering and thus becomes a significant factor when considering model analysis (Xu et al., 2021). Although human activity contributes to the generation of extra protons, the water pH measured was mildly alkaline, suggesting that proton contributions from anthropogenic source may accelerate chemical weathering. Human activities mainly affect Na^+^, Cl^−^, K^+^, NO_3_^−^ and SO_4_^2−^. We had already discussed the spatial variability of these ions and found that the concentration of these parameters was much higher in the middle-lower basin than that in the upper part, indicating strong impact of urban and agricultural inputs. For anthropogenic-originated chlorine, the Cl_anth_ were obtained by subtracting the minimum value from riverine Cl. For K and Na, the Cl^−^-normalized ratios in urban wastewater (K/Cl = 0.3, Na/Cl = 0.2) (Chetelat et al., 2008) were applied to be the references of human activities. Then, after the atmospheric and anthropogenic input correction, the remainder of K and Na were seen from the source of silicate weathering, since these two were generally obtained from feldspar. However, due to complex lithology settings in the JLJ River basin, the accurate measurements of Ca_sili_ and Mg_sili_ were hindered from the straightforward method of data processing to a certain extent. Therefore, the generally accepted average Na-normalized values of Ca and Mg in silicate bedrocks from literatures were applied to this study (Ca/Na = 0.43, Mg/Na = 0.26) (Wang et al., 2015; Zhang et al., 2012).

Due to seasonal variations in riverine discharge, we assumed that discharge in winter (dry season) accounts for 25% of the discharge in the JLJ River basin in the whole year. Therefore, the silicates weathering rates (SWR) can be calculated by cations from silicate weathering (either by H_2_CO_3_ or H_2_SO_4_) based on Eq. (2). The results of silicate weathering rates in dry season exhibited relatively large variations from 10.9 to 27.5 t km^−2^ per year within the JLJ River basin. As shown in Table 3, the highest SWR appeared in tributary WR (27.5 t km^−2^ per year), nearly three times higher than that in the mainstream NR (10.9 t km^−2^ per year) and 1.5 times higher than that in SR. Silicate weathering rates was calculated to be 55.7 t km^−2^ per year in total. Furthermore, compared to the mean silicate weathering rate of 23.7 t km^−2^ per year in the SE coastal river basin (Liu et al., 2018), the SWR of the JLJ River basin is relatively higher even though in the dry season. This phenomenon is probably due to different climate conditions in this two regions, since lower runoff and longer residence time of river in dry season could smooth variations and aggravate silicate weathering to some extent.

**Table 3:** Silicate weathering and CO_2_ consumption rates of the JLJ River basin.

Parameters	Units	Mainstream (NR)		Tributary (WR)		Tributary (SR)		Total
		Annual average	Dry season	Annual average	Dry season	Annual average	Dry season	
Discharge	m^3^ s^−1^	266.8	122.4	126.7	66.1	70.6	17.7	464.2
Na_sili_	mg L^−1^		6.4		17.0		20.8	
K_sili_	mg L^−1^		1.8		1.6		—	
Ca_sili_	mg L^−1^		2.8		7.3		5.4	
Mg_sili_	mg L^−1^		1.7		4.4		9.0	
SiO_2_	mg L^−1^		11.4		19.0		23.0	
River area	km^2^		8,490		3,737		1,873	14,100
SWR in JLJ basin	t km^−2^ y^−1^		10.9		27.5		17.3	55.7
ФCO_2_-Sili in JLJ basin	10^5^ mol km^−2^ y^−1^		2.74		8.44		5.71	16.9
World average ФCO_2_-Sili	10^5^ mol km^−2^ y^−1^							1
CO_2_ sink in JLJ basin	10^−6^ Gt C y^−1^		102.25		138.83		47.07	288.15
Terrestrial CO_2_ sink	Gt C y^−1^							3.0 ± 0.8
CO_2_ sink in ocean	Gt C y^−1^							2.4 ± 0.5

**Note:**

“—” means that the calculated value is negative and is managed it by zero.

The results of CO_2_ consumption rate were calculated based on Eq. (3) and exhibited in Table 3. According to our calculation, the value of ФCO_2_-Sili in dry season varied from 2.74 × 10^5^ to 8.44 × 10^5^ mol km^−2^ per year within the JLJ basin, slightly higher than world average (1 × 10^5^ mol km^−2^ per year) (Gaillardet et al., 1999). Assuming the consistency of SWR on continental surface, silicate weathering derived-CO_2_ sink was estimated to be 288.15 × 10^−6^ Gt C per year in dry season. Although this value seems to be much smaller than that in terrestrial biosphere (3.0 ± 0.8 Gt C y^−1^) and ocean (2.4 ± 0.5 Gt C y^−1^) (Le Quéré et al., 2017), the CO_2_ sink derived from silicate weathering may still play an essential role in coastal carbon budget. The various additional protons supplied by anthropogenic inputs would alter the weathering cycle of dissolved loads in the coastal river basin, consequently change the chemical balance of dissolved ions in the adjacent ocean through river output flux, and make continuous contribution to seawater Sr evolution in the long run.

### Dissolved Sr flux and the impact on oceanic system

Quantitatively estimating the dissolved Sr flux in the JLJ River can provide identical insights into the transport of strontium from rivers to oceans, since JLJ River flows through Xiamen Bay into the East China Sea eventually. According to previous research, no obvious variation of annual river runoff was found in our study region (Huang, 2008). The river discharge in the JLJ mainstream and tributaries were derived from our previous work (Liu et al., 2019a). In detail, the river discharge data from hydrologic station Punan (located on the mainstream) and Zhengdian (on the tributary WR) were chosen to represent the average discharge of NR (122.44 m^3^ s^−1^) and WR (66.12 m^3^ s^−1^) in dry season, respectively (Table 4). Considering that there isn’t any hydrological station on tributary SR, the annual mean discharge of SR (70.6 m^3^ s^−1^, 15.2%) could be estimated by subtracting that of NR (266.8 m^3^ s^−1^, 57.5%) and WR (126.7 m^3^ s^−1^, 27.3%) from the annual average runoff in the whole river basin (464.2 m^3^ s^−1^) (Huang, 2008; Liu et al., 2019a).

**Table 4:** River runoff and the flux of dissolved Sr in the JLJ River basin.

Parameters	Units	Mainstream (NR)		Tributary (WR)		Tributary (SR)	Total
		Annual average	Dry season	Annual average	Dry season	Annual average	
River runoff (Q_JLJ_)	m^3^ s^−1^	266.8	122.44	126.7	66.12	70.6	464.2
Dissolved Sr content (C_Sr_)	mg L^−1^	0.029	0.006	0.030			
Dissolved Sr flux (F_Sr_)	t y^−1^	559.9	112.0	62.6	12.5	66.8	689.2
Dissolved Sr flux (F_Sr_)	mol y^−1^	6,362,316	1,272,463	7,10,850	1,42,170	7,59,014	7,832,181
^87^Sr_excess_ flux	t y^−1^						17.3

Data from the sampling site N23 (0.029 mg L^−1^), W10 (0.006 mg L^−1^) and S4 (0.030 mg L^−1^) were selected to represent the identical dissolved Sr contents of the mainstream (NR) and tributary (WR), as they were situated in the most downstream locations without tidal influence. In addition, for the sake of simplification in our calculation to a certain degree, we assumed the constancy of the values measured during sampling period, and that the discharge in dry season occupied about 20% of the total yearly discharge in the JLJ river based on generally accepted experience. Besides, we did not calculate the seasonal values of SR due to lack of hydrological data in SR, and instead estimate the yearly riverine Sr flux of SR based on the figures obtained above. As presented in Table 4, the Sr flux towards the East China Sea was calculated to be 689.2 t per year based on Eq. (4) with the ^87^Sr/^86^Sr of 0.7341 (Site E1), much higher than the dissolved Sr flux estimated in previous study (355.4 t yr^−1^) conducted 10 years ago in 2008 (Zhou et al., 2012), indicating accelerated transportation of dissolved Sr over time from coastal river to the adjacent ocean, which may also contribute to the potential impact on oceanic system. Going further, the dissolved Sr flux to the ocean in the JLJ dry season is about 112.0 t y^−1^ for the mainstream (NR) and 12.5 t y^−1^ for the WR tributary, which indicating that the mainstream (NR) contributes 10 times more of the dissolved Sr flux relative to the WR.

Moreover, in order to identify the flux of ^87^Sr in excess of the generally consistent oceanic Sr isotopic composition (0.709), the ^87^Sr_excess_ flux was calculated based on the following equation (Bickle et al., 2003):

(6) $${}_{}^{{\rm 87}}{\rm S}{{\rm r}_{{\rm excess}}}{\rm flux} = \left( {\displaystyle{{{}_{}^{{\rm 87}}{\rm Sr}} \over {{}_{}^{{\rm 86}}{\rm Sr}}}{\rm \; - \; 0}{\rm .709}} \right){\rm \times \; Total\; S}{{\rm r}_{{\rm flux}}}$$

The calculated ^87^Sr flux in excess is about 17.3 t y^−1^ (0.2 × 10^6^ mol y^−1^) in the JLJ River (Table 4), indicating an essential impact of the JLJ River on seawater Sr isotope evolution.

## Conclusions

This paper focused on the strontium isotopes and hydro-chemistry of the Jiulongjiang River in tracing natural inputs and human activities on dissolved loads to the adjacent ocean. The Sr content and ^87^Sr/^86^Sr ratios were 0.07–0.90 μmol L^−1^ and 0.7140–0.7514, respectively. Silicate weathering accounted for more than half of total Sr and exhibited 45.7–69.8% to the estuary, implying the predominant role of silicate weathering. The silicate-derived Sr flux to the adjacent ocean was 689.2 t y^−1^, implying an essential impact of coastal watershed on seawater Sr isotope evolution. The silicate weathering rate and CO_2_ consumption rate were estimated to be 10.9–27.5 t km^−2^ per year and 2.74–8.44 × 10^5^ mol km^−2^ per year in dry season, respectively, mildly higher than world average, implying that lower runoff and longer residence time of river in dry season may aggravate silicate weathering. Although the CO_2_ sink by silicate weathering in JLJ river seems smaller than that in main reservoirs, it may play an essential role in coastal carbon budget. Human inputs could accelerate chemical weathering through the addition of strong acids. Various sources and protons participated in silicate weathering process would alter weathering cycle of dissolved loads in the coastal watershed, and with the addition of anthropogenic inputs, consequently alter chemical balance in the adjacent ocean through river flux, and make continuous contribution to seawater Sr evolution in the long run.

## Supplemental Information

10.7717/peerj.13223/supp-1 Supplemental Information 1 Raw data.

10.7717/peerj.13223/supp-2 Supplemental Information 2 The corresponding test statistics and the exact p-value of the Pearson correlation analysis.

10.7717/peerj.13223/supp-3 Supplemental Information 3 The descriptive statistics of the detected parameters.

## Additional Information and Declarations

### Data Availability

The following information was supplied regarding data availability: The raw measurements are available in the Supplemental Files.
