# Supplementary material for: Geochemical characteristics of strontium isotopes in a coastal watershed: implications for anthropogenic influenced chemical weathering and export flux
Source: PeerJ. 2022 Apr 5;10:e13223. doi: 10.7717/peerj.13223 (PMC8992644; doi:10.7717/peerj.13223)
Supplement: Supplemental Information 2 [file peerj-10-13223-s002.docx]

|  | pH | EC | Na^+^ | K^+^ | Ca^2+^ | Mg^2+^ | Cl^-^ | NO_3_^-^ | SO_4_^2-^ | HCO_3_^-^ | SiO_2_ | TDS | Sr |  |
| --- | --- | --- | --- | --- | --- | --- | --- | --- | --- | --- | --- | --- | --- | --- |
| pH | 1 |  |  |  |  |  |  |  |  |  |  |  |  |  |
| **Sig.** |  |  |  |  |  |  |  |  |  |  |  |  |  |  |
| EC | 0.173 | 1 |  |  |  |  |  |  |  |  |  |  |  |  |
| **Sig.** | **0.31** |  |  |  |  |  |  |  |  |  |  |  |  |  |
| Na^+^ | 0.057 | 0.836** | 1 |  |  |  |  |  |  |  |  |  |  |  |
| **Sig.** | **0.74** | **0.00** |  |  |  |  |  |  |  |  |  |  |  |  |
| K^+^ | 0.082 | 0.740** | 0.808** | 1 |  |  |  |  |  |  |  |  |  |  |
| **Sig.** | **0.64 0.000** | **0.00** | **0.00** |  |  |  |  |  |  |  |  |  |  |  |
| Ca^2+^ | 0.333* | 0.841** | 0.454** | 0.396* | 1 |  |  |  |  |  |  |  |  |  |
| **Sig.** | **0.05** | **0.00** | **0.01** | **0.02** |  |  |  |  |  |  |  |  |  |  |
| Mg^2+^ | 0.283 | 0.912** | 0.608** | 0.517** | 0.911** | 1 |  |  |  |  |  |  |  |  |
| **Sig.** | **0.10** | **0.00** | **0.00** | **0.00** | **0.00** |  |  |  |  |  |  |  |  |  |
| Cl^-^ | 0.099 | 0.821** | 0.963** | 0.762** | 0.460** | 0.636** | 1 |  |  |  |  |  |  |  |
| **Sig.** | **0.56** | **0.00** | **0.00** | **0.00** | **0.01** | **0.00** |  |  |  |  |  |  |  |  |
| NO_3_^-^ | 0.038 | 0.477** | 0.424* | 0.789** | 0.281 | 0.382* | 0.389* | 1 |  |  |  |  |  |  |
| **Sig.** | **0.83** | **0.00** | **0.01** | **0.00** | **0.10** | **0.02** | **0.02** |  |  |  |  |  |  |  |
| SO_4_^2-^ | 0.108 | 0.731** | 0.377* | 0.208 | 0.848** | 0.814** | 0.348* | 0.101 | 1 |  |  |  |  |  |
| **Sig.** | **0.53** | **0.00** | **0.02** | **0.22** | **0.00** | **0.00** | **0.04** | **0.56** |  |  |  |  |  |  |
| HCO_3_^-^ | 0.247 | 0.673** | 0.453** | 0.316 | 0.742** | 0.658** | 0.447** | -0.002 | 0.451** | 1 |  |  |  |  |
| **Sig.** | **0.15** | **0.00** | **0.01** | **0.06** | **0.00** | **0.00** | **0.01** | **0.98** | **0.01** |  |  |  |  |  |
| SiO_2_ | -0.073 | 0.244 | 0.517** | 0.609** | -0.149 | 0.039 | 0.456** | 0.465** | -0.362* | 0.185 | 1 |  |  |  |
| **Sig.** | **0.67** | **0.15** | **0.00** | **0.00** | **0.39** | **0.82** | **0.01** | **0.00** | **0.03** | **0.28** |  |  |  |  |
| TDS | 0.174 | 0.990** | 0.841** | 0.787** | 0.824** | 0.899** | 0.817** | 0.565** | 0.685** | 0.653** | 0.318 | 1 |  |  |
| **Sig.** | **0.31** | **0.00** | **0.00** | **0.00** | **0.00** | **0.00** | **0.00** | **0.00** | **0.00** | **0.00** | **0.059** |  |  |  |
| Sr | 0.053 | -0.21 | -0.174 | 0.005 | -0.227 | -0.138 | -0.147 | 0.207 | -0.257 | -0.336* | 0.071 | -0.179 | 1 |  |
| **Sig.** | **0.76** | **0.22** | **0.31** | **0.98** | **0.18** | **0.42** | **0.39** | **0.23** | **0.13** | **0.05** | **0.68** | **0.30** |  |  |
| Note: *Correlation is significant at *p* < 0.05 level; **Correlation is significant at *p* < 0.01 level; Sig. means the significant difference (p-value). | | | | | | | | | | | | | | |
