# Supplementary material for: Geochemical characteristics of strontium isotopes in a coastal watershed: implications for anthropogenic influenced chemical weathering and export flux
Source: PeerJ. 2022 Apr 5;10:e13223. doi: 10.7717/peerj.13223 (PMC8992644; doi:10.7717/peerj.13223)
Supplement: Supplemental Information 3 [file peerj-10-13223-s003.docx]

|  | **Mean value** | **Standard Deviation** |
| --- | --- | --- |
| **pH** | 7.18 | 0.438 |
| **EC** | 210.28 | 96.589 |
| **Na^+^** | 9.98 | 6.713 |
| **K^+^** | 4.74 | 2.183 |
| **Ca^2+^** | 15.23 | 7.305 |
| **Mg^2+^** | 2.75 | 1.510 |
| **Cl^-^** | 9.76 | 9.204 |
| **NO_3_^-^** | 12.79 | 10.636 |
| **SO_4_^2-^** | 18.06 | 14.306 |
| **HCO_3_^-^** | 37.28 | 14.983 |
| **SiO_2_** | 15.13 | 5.048 |
| **TDS** | 107.05 | 44.552 |
| **Sr** | 0.03 | 0.015 |
